# A new prediction model for operative time of flexible ureteroscopy with lithotripsy for the treatment of renal stones

**DOI:** 10.1371/journal.pone.0192597

**Published:** 2018-02-13

**Authors:** Shinnosuke Kuroda, Hiroki Ito, Kentaro Sakamaki, Tadashi Tabei, Takashi Kawahara, Atsushi Fujikawa, Kazuhide Makiyama, Masahiro Yao, Hiroji Uemura, Junichi Matsuzaki

**Affiliations:** 1 Department of Urology, Ohguchi Higashi General Hospital, Yokohama, Japan; 2 Department of Urology and Renal Transplantation, Yokohama City University Medical Center, Yokohama, Japan; 3 Department of Urology, Yokohama City University Hospital, Yokohama, Japan; 4 Department of Biostatistics, Yokohama City University Graduate School of Medicine, Yokohama, Japan; Sun Yat-sen University, CHINA

## Abstract

This study aimed to develop a prediction model for the operative time of flexible ureteroscopy (fURS) for renal stones. We retrospectively evaluated patients with renal stones who had been treated successfully and had stone-free status determined by non-contrast computed tomography (NCCT) 3 months after fURS and holmium laser lithotripsy between December 2009 and September 2014 at a single institute. Correlations between possible factors and the operative time were analyzed using Spearman’s correlation coefficients and a multivariate linear regression model. The P value < 0.1 was used for entry of variables into the model and for keeping the variables in the model. Internal validation was performed using 10,000 bootstrap resamples. Flexible URS was performed in 472 patients, and 316 patients were considered to have stone-free status and were enrolled in this study. Spearman’s correlation coefficients showed a significant positive relationship between the operation time and stone volume (ρ = 0.417, p < 0.001), and between the operation time and maximum Hounsfield units (ρ = 0.323, p < 0.001). A multivariate assessment with forced entry and stepwise selection revealed six factors to predict the operative time of fURS: preoperative stenting, stone volume, maximum Hounsfield unit, surgeon experience, sex, and sheath diameter. Based on this finding, we developed a model to predict operative time of fURS. The coefficient of determination (R^2^) in this model was 0.319; the mean R^2^ value for the prediction model was 0.320 ± 0.049. To our knowledge, this is the first report of a model for predicting the operative time of fURS treatment of renal stones. The model may be used to reliably predict operative time preoperatively based on patient characteristics and the surgeons’ experience, plan staged URS, and avoid surgical complications.

## Introduction

According to the latest guidelines, treatment recommendations for urolithiasis have shifted to endourologic procedures, such as flexible ureteroscopy (fURS) and percutaneous nephrolithotomy (PCNL). Consequently, shock wave lithotripsy (SWL) has lost its place as the first-line modality for many indications. Flexible URS has become a standard treatment for renal and ureteral calculi less than 20 mm [1]. Given the advancement in surgical techniques and devices in fURS, successful outcomes and low complication rates have been reported, even in complicated cases, such as solitary kidney patients [2–6]. However, operative time can influence surgical outcomes, especially complications in the fURS procedure [7]. In endourological surgeries, such as URS or PCNL, several severe perioperative and postoperative complications can occur, including sepsis, perforation, and massive bleeding [8–11]. A previous study reported that the severe adverse events after URS were associated with a longer operative time and lower number of the URS surgeries being performed at the hospital [12]. Therefore, it is important to understand the clinical preoperative parameters that prolong the operative time of fURS procedures.

We have previously reported the significant parameters that aid in predicting the operative time of fURS, such as stone volume, experience level of the surgeon, Hounsfield units (HUs), and preoperative stenting [13]. However, a prediction model for precise operative time using these parameters has not yet been developed. A prediction model may help the surgeon plan the operation more precisely, predict the requirement for additional fURS sessions, allow the patients to be better informed, and avoid surgical complications.

In this study, we aimed to develop a prediction model for operative time of fURS and propose a prediction model based on the preoperative clinical parameters.

## Materials and methods

### Patient data

Total 472 fURS procedures were retrospectively analyzed in this study and all procedures were performed for renal stone treatment between December 2009 and December 2014 at Ohguchi East General Hospital. Multi-stage fURS were excluded in this study to exclude potential factors that could affect the operative time of fURS. Stone-free status (SF) was assessed 3 months after the fURS using non-contrast computed tomography (NCCT) and was defined as no visible stones. To assess the operative time predictability of each clinical parameter, the procedures resulting in SF following fURS were examined. The procedures resulting in non-SF after fURS were excluded, because these failed procedures did not enable us to measure the precise stone volume treated successfully during fURS.

The present study was carried out under the Ethics Committee of Ohguchi East General Hospital (No. H25-3169). Written informed consent from all patients were obtained for their data to be used for research purposes.

### Surgical techniques

Surgical techniques were described in our previous literatures [13, 14]. Briefly, flexible URS with lithotripsy (200-μm Holmium: yttrium-aluminum-garnet laser) was performed with a 6/7.5 or 8/9.8 Fr semi-rigid ureteroscope and a flexible ureteroscope. All enrolled procedures were carried out with placement of ureteral access sheaths (12/14 or 14/16 Fr, Cook Medical, Bloomington, IN or 11/13 or 13/15 Fr, Boston Scientific, Natick, MA) to reduce the intrarenal pressure and facilitate stone extraction. To avoid unnecessary surgical complications, in case that the operative time exceeded 120 min, we discontinued the procedure.

### Preoperative and postoperative evaluations

Patient’s backgrounds and stone characters were analyzed including stone volume (mm^3^), stone number, maximum and mean HUs, preoperative placement of ureteral stent, diameter of ureteral access sheath and operative time. Stone volume was determined on 5 mm axial and 3.5 mm reconstructed coronal NCCT, as previously described [14]. The number of stones, maximum and mean HUs [15], presence of hydronephrosis, and lower pole calculi were evaluated by preoperative NCCT. Ten urologists in total were involved in this study and all surgical techniques were almost identical as nine urologists were supervised by same expert (J.M.) until they obtain proper surgical techniques, and then followed same procedure protocols.

### Development of prediction model of fURS surgical time

Correlations between possible factors and the operative time were analyzed using a multivariate linear regression model with stepwise selection. Linear regression is an approach for modeling the relationship between a scalar dependent variable and the other independent variables, which can be used to fit a predictive model [16]. A total of 11 clinical factors were enrolled in the multivariate linear regression analysis. On the basis of our previous reports [13], 4 of the 11 clinical factors, including preoperative stenting, stone volume, HU, and surgeon’s experience, were directly enrolled in the linear regression model with the forced entry method. For the other 7 clinical factors, P < 0.1 was used for entry into the model and for keeping the variables in the model with stepwise selection [17]. The predictive ability of the resulting extended models was expressed by the model’s coefficient of determination (R^2^) [16]. The finally selected model was used as the prediction model. Internal validation was performed using 10,000 bootstrap resamples. Statistical analyses were performed using SAS 9.3 (SAS Institute Inc., Cary, NC).

### Statistical analysis

Statistical analysis was performed with the Statistical Package for Social Sciences, version 21 (SPSS, Chicago, IL). All data are expressed as mean ± standard error of the mean (SEM). Patient characteristics and preoperative factors were analyzed using the unpaired t- and Chi-square tests. The correlation between the factors and the operative time was analyzed using Spearman’s coefficient values.

## Results

Among the 472 patients in whom fURS procedures were performed, 316 (66.9%) were considered to have SF status following fURS (S1 File). Table 1 shows the comparison of patient and stone characteristics among the procedures with different outcomes. The patients who had non-SF status were divided into the following two groups: residual fragments (RF) less than 4 mm and RF more than 4 mm. Significant differences were found between SF and RF less than 4 mm with respect to the following parameters: In the SF group, stone volume was smaller (651.6 mm^3^ vs. 1286.8 mm^3^, P = 0.002), there were fewer stones in the lower pole calculi (57.0% vs. 84.7%, P < 0.001), both maximum and mean HUs were lower (1150.4 HU vs. 1298.4 HU; P = 0.022, 937.4 HU vs. 1077.3 HU; P = 0.015, respectively), and operative time was shorter (P < 0.001). In addition to the parameters above, the number of stones (2.7 vs. 3.3, P < 0.001) and access sheath diameter (P < 0.001) were significantly different in the SF group than in the group with RF more than 4 mm.

**Table 1:** Comparison of patient and stone characteristics among the procedures with stone-free status, residual fragments less than 4mm and residual fragments more than 4mm.

			Operative outcome			P value		
			Stone free status (SF) ^#1^	Residual fragments (RF)		#1 vs #2	#1 vs #3	#2 vs #3
				< = 4mm ^#2^	> 4mm ^#3^			
No. of patients (cases)			316	59	97			
Age (years)			60.1 ± 13.6	59.2 ± 13.0	59.5 ± 13.8	N.S.	N.S.	N.S.
Sex	female (cases)		126	31	41	0.070	N.S.	N.S.
	male (cases)		190	28	56			
Side	rt (cases)		132	23	43	N.S.	N.S.	N.S.
	lt (cases)		182	36	54			
Body mass index (cm/kg2)			24.1 ± 4.3	23.3 ± 3.8	23.1 ± 4.2	N.S.	N.S.	N.S.
No. of stones			2.0 ± 1.8	2.7 ± 1.9	3.3 ± 2.5	0.051	< 0.001	N.S.
Stone volume (mm3)			651.6 ± 690.5	1286.8 ± 1122.0	3725.9 ± 4354.5	0.002	< 0.001	< 0.001
Lower pole calculi (cases)			180	50	81	< 0.001	< 0.001	N.S.
Hounsfield unit	maximum		1150.4 ± 419.0	1298.4 ± 360.0	1378.6 ± 313.9	0.022	< 0.001	N.S.
	mean		937.4 ± 363.3	1077.3 ± 361.9	1110.5 ± 310.5	0.015	< 0.001	N.S.
Hydronephrosis (cases)			162	31	55	N.S.	N.S.	N.S.
Access sheath	diameter	9.5 Fr	8	1	5	N.S.	< 0.001	< 0.001
		11 Fr	141	15	15			
		12 Fr	54	15	14			
		13 Fr	56	16	14			
		14 Fr	57	12	49			
Preoperative stenting (cases)			164	28	51	N.S.	N.S.	N.S.
SWL failure (cases)			79	16	24	N.S.	N.S.	N.S.
Operative time (min)			80.3 ± 33.7	109.2 ± 34.4	110.5 ± 23.0	< 0.001	< 0.001	N.S.
Operator	> = 50 URS performed		199	39	68	N.S.	N.S.	N.S.
	< 50 URS performed		117	20	29			
Postoperative admission days			3.7 ± 2.2	3.9 ± 3.3	4.1 ± 2.1	N.S.	N.S.	N.S.
Postoperative fever (cases)			13	2	7	N.S.	N.S.	N.S.
Postoperative ureteral stricture (cases)			2	0	0	N.S.	N.S.	N.S.

SWL: shock wave lithotripsy; URS: ureterorenoscopy.

High-grade fever occurred in 21 patients. All patients were treated conservatively (Clavien Classification Grade II). Ureteral stricture developed in two patients with SF status. In both cases, no obvious perioperative problems were identified. Balloon dilation of the ureter was performed in two cases, which resulted in a successful outcome in one patient; balloon dilation failed in the other patient and placement of permanent double-J stents was performed (Clavien Classification Grade III). There was no significant difference between patients with SF status and those with non-SF status with regard to complications.

**Fig 1:**
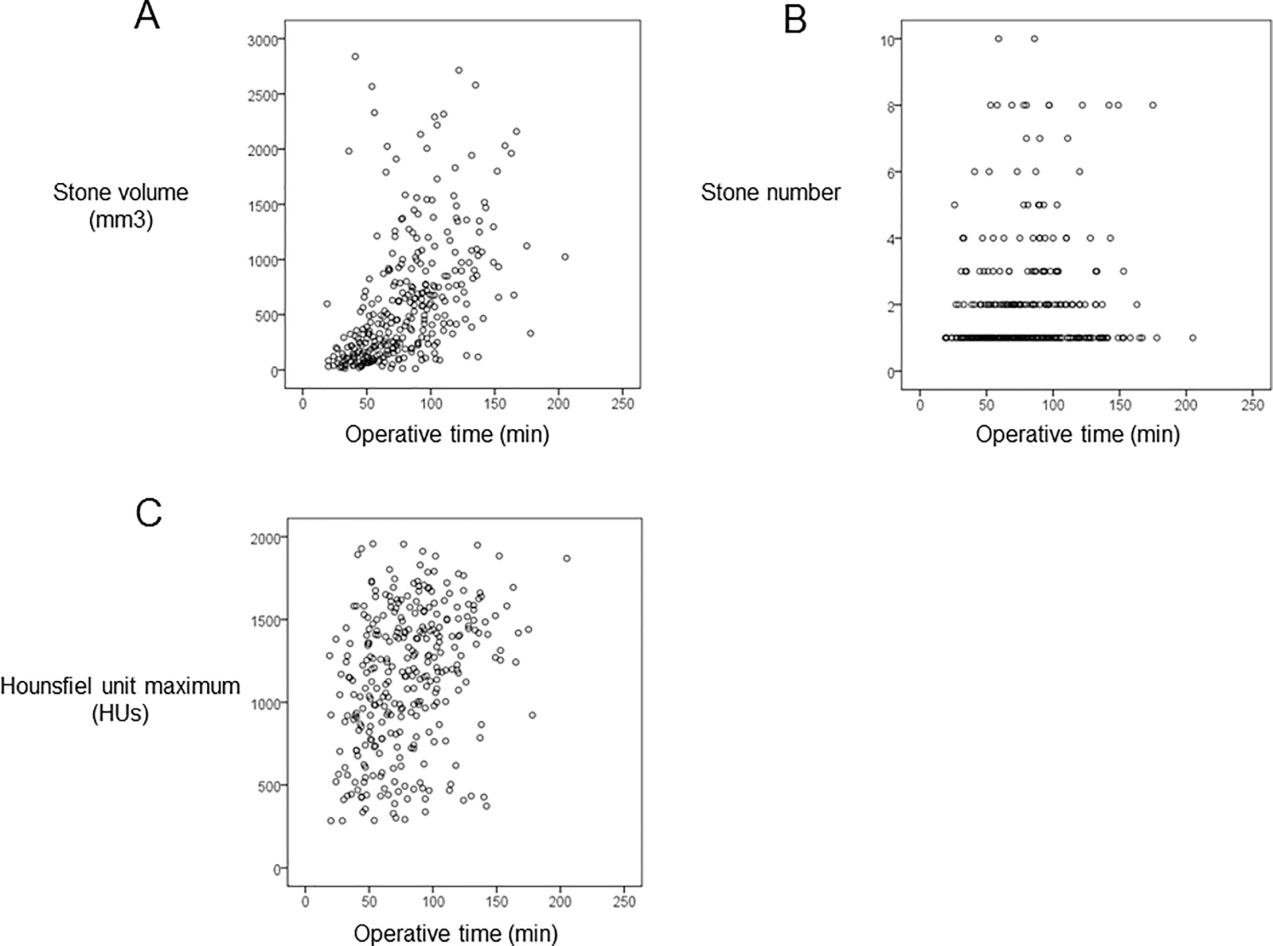
The scatter plots of correlation between operation time of fURS and stone number, stone volume, and maximum HU. A significant positive correlation was found between the operation time and stone volume (ρ = 0.417, P < 0.001) and between operation time and HU (ρ = 0.323, P < 0.001).

Among the 316 SF procedures, the correlation between the operation time of fURS and each parameter was analyzed. The scatter plots of correlation between operation time of fURS and stone number, stone volume, and maximum HU are shown in Fig 1. A significant positive correlation was found between the operation time and stone volume (ρ = 0.417, P < 0.001) and between operation time and HU (ρ = 0.323, P < 0.001) (shown in Table 2).

**Table 2:** Coefficient values between the operative time of flexible ureteroscopy with lithotripsy and the stone volume, stone number and Hounsfield unit maximum.

	Spearman's coefficient values	P value
Stone volume (mm3)	0.417	< 0.001
Stone number	0.108	0.054
Hounsfield unit maximum	0.323	< 0.001

Multivariate linear regression analysis with forced entry and stepwise selection was performed using the 316 procedures in SF groups. The analysis developed three models of variables for predicting the operation time of fURS (Table 3). Coefficient of determination (R^2^) of the model was 0.319. Model 3 leads the formula of predicting operating time of fURS as follows:

Operative time of fURS for renal stone treatment (min)

= **43.60** (Constant)
+ (stone volume (mm^3^) × **0.02**)
+ (maximum HU × **0.02**)
+ (**16.56** if operator experience is less than 50 cases)
+ (**10.44** if male)
- (**4.68** if presence of preoperative stenting)
- (ureteral sheath diameter (Fr) × **3.57**).

**Table 3:** Summary of linear regression model for variables predicting the operative time of flexible ureteroscopy with lithotripsy for the treatment of renal stone (N = 316).

	Model				
Valuable	B	B SE	β	P value	95% COI for B
(Constant)	43.60	21.72			
Preoperative stenting	4.68	3.31	0.07	0.158	-1.83–11.19
Stone volume	0.02	0.00	0.41	< 0.001	0.02–0.03
Hounsfield unit maximum	0.02	0.00	0.22	< 0.001	0.01–0.03
Operator experience	16.56	3.46	0.23	< 0.001	9.75–23.37
Sex	10.44	3.41	0.15	0.002	3.73–17.14
Sheath diamater	-3.57	1.54	-0.13	0.021	-6.60 - -0.54
R^2^	0.319				

B: Unstandardized coefficients.

B SE: Standard error of unstandardized coefficients.

β: Standardized coefficients.

R^2^: Coefficient of determination.

Internal validation of the model showed that the average coefficient of determination was 0.320 ± 0.049 (0.160–0.503), which showed that there was no overfitting in this model.

## Discussion

Successful and eligible outcomes of fURS have been well demonstrated, even in cases of large and multiple renal stones, and show low complication and morbidity rates [2–6]. Nonetheless, severe complications (septic shock, cardiovascular events, and blood loss) could still be associated with fURS; a longer operative time is one of the crucial risk factors of these complications [11]. Sugihara et al. reported that adverse events significantly increased when the operation time exceeded 90 min [12]. Thus, especially in cases of large or multiple stones, it is important to accurately predict operative time and arrange the operative plan more precisely so that we can achieve SF within a reasonable operative time. In the present study, we have developed the prediction model of operation time based on preoperative clinical factors. The model consists of six clinical factors: stone volume, maximum HUs, operator experience, sex, preoperative stenting, and ureteral sheath diameter.

Stone volume was the most significant parameter that prolonged operative time. This was a conceivable and acceptable result considering the basic principle of fURS. Previous studies have shown that stone burden, especially stone volume, is the most important predictor of SF following URS [14, 18]. Therefore, accurate preoperative calculation of stone volume by NCCT is essential to predict not only the operative outcome, but also the operative time of fURS procedures.

The benefits of preoperative stenting have been evaluated, and some studies suggest that ureteral stent placement before URS can passively dilate the ureter, enabling easier removal of stones, which shortens operative time [19, 20]. Previously, Chu et al. showed that preoperative stenting decreased the operative time of URS procedures using non-multivariate statistical analysis [21]. Although stenting offers significant benefits to the operation time in fURS, there is no previous report showing a significant relationship between the operation time and the diameter of ureteral access sheath. Torricelli et al. compared the data from patients who underwent fURS with and without preoperative stenting with operative outcomes [22]. They reported that the stented group had larger ureteral access sheaths (P < 0.001). It is expected that it is easier to remove a larger stone fragment with lower intrarenal pressure under a larger ureteral access sheath. In our study, with statistical significance, we prove that a larger access sheath contributes to shortening of the operative time of fURS. However, the use of a ureteral access sheath also carries the risk of acute ureteral injury related to insertion, and ischemia of the ureter [23]. Guzelburc et al. reported intraoperative ureteral injuries during a retrograde intrarenal surgery with a ureteral access sheath using the Post-Ureteroscopic Lesion Scale; low grade injuries were found in 41.5% of the patients [24]. The use of a larger ureteral access sheath may increase the risk of these injuries and ischemia; therefore, we need to consider both the risk and the benefits of a larger ureteral access sheath in each case.

A previous study has suggested that HUs measured by NCCT can predict the fragility of urinary stones [25], and other clinical studies have indicated that HUs measured by NCCT can predict the efficacy of SWL [26, 27]. Our group also previously reported that higher HUs, as measured by NCCT, prolonged operative time in fURS [15]. This is because HUs predict the stone hardness and fragility. In our previous study, we also proved that HUs could predict stone composition through a multivariate analysis [28]. Considering clinical use, it is easy to measure HUs using NCCT; however, it is usually difficult to predict the stone composition preoperatively.

The experience of the operator showed a significant association with the operative time of fURS procedures. In our institution, operators with an experience of fewer than 50 fURS procedures had longer operative times. The clinical nomograms to predict SF following fURS developed in our institution showed that operator experience of fewer than 50 fURS procedures was associated with higher levels of treatment failure [29]. Thus, it is clear that operator experience is associated with both operative time and quality of outcome.

Our study showed that operative time is shorter if the patient is female. To our knowledge, this is the first study to highlight the influence of sex on the operative time of fURS. One possible reason is that the distance between the stones and the external urethral meatus is shorter if the patient is female because the length of the urethra is longer in males. If the stone is in the renal pelvis or calyx in male patients, usually we need to use 46 cm or longer ureteral access sheath; it is unusual to use this sheath size in female patients. Therefore, easier access to the stones is likely to make the operation time shorter in female patients, especially during stone removal.

This prediction model will help the surgeon plan the operation more precisely. In case the operation time is presumed to be more than 120 min, staged fURS or PCNL would be the recommended option due to the complications associated with a longer time, even if the size of stone is less than 20 mm. The model will also enable us to provide patients with more detailed information about fURS.

There are some limitations to this study. First, the study was based on retrospective patient data from a single institute. Multicenter analysis will be needed for further research. Second, this study assessed SF procedures, which might mean that the findings were suitable for applying to procedures with successful outcomes, because it was difficult to measure the precise stone volume that was successfully treated with fURS in the case of unsuccessful procedures.

## Conclusions

We have developed a model to predict the operative time during fURS, and to our knowledge, this is the first study to report such a model. This model utilizes 6 preoperative characteristics: stone volume, maximum HUs, operator experience, sex, preoperative stenting, and ureteral sheath diameter. The model may be used to reliably predict operative time preoperatively based on patient characteristics and surgeons’ experience of the procedure, plan staged URS, and avoid surgical complications.

## Supporting information

S1 File The data sheet of clinical data analysed in the study. Data lists of analyzed clinical parameters.
(XLS)
